# Water Supply Challenges in Rural Areas: A Case Study from Central Kazakhstan

**DOI:** 10.3390/ijerph16050688

**Published:** 2019-02-26

**Authors:** Alua Omarova, Kamshat Tussupova, Peder Hjorth, Marat Kalishev, Raushan Dosmagambetova

**Affiliations:** 1 Department of Public Health, Karaganda Medical University, Gogol Street 40, Karaganda 100008, Kazakhstan; alua_1912@mail.ru (A.O.); kalishev@kgmu.kz (M.K.); dosmagambetova@kgmu.kz (R.D.); 2 Division of Water Resources Engineering, Lund University, Box 118, SE-221 00 Lund, Sweden; peder.hjorth@tvrl.lth.se; 3 Center for Middle Eastern Studies, Lund University, Box 221, SE-221 00 Lund, Sweden

**Keywords:** access to water, drinking water sources, perceived water quality, reliability of water supply systems, rural area, volume of water consumption

## Abstract

Rural water supplies have traditionally been overshadowed by urban ones. That must now change, as the Sustainable Development Goals calls for water for all. The objective of the paper is to assess the current access to and the perceived water quality in villages with various types of water supply. The survey was carried out during July–December 2017 in four villages in central Kazakhstan. Overall, 1369 randomly selected households were interviewed. The results revealed that even though villagers were provided with tap water, significant numbers used alternative sources. There were three reasons for this situation: residents’ doubts regarding the tap water quality; use of other sources out of habit; and availability of cheaper or free sources. Another problem concerned the volume of water consumption, which dropped sharply with decreased quality or inconvenience of sources used by households. Moreover, people gave a poor estimate to the quality and reliability of water from wells, open sources and tankered water. The paper suggests that as well decentralization of water management as monitoring of both water supply and water use are essential measures. There must be a tailor-made approach to each village for achieving the Sustainable Development Goal of providing rural Kazakhstan with safe water.

## 1. Introduction

The target task of the Millennium Development Goal (MDG) 7.C was to halve the number of the population with no access to safe drinking water and basic sanitary facilities by the year 2015 [1,2,3]. Through implementing this target, the proportion of people who have access to a basic drinking water service grew from 81% to 89% from 2000 to 2015 [4,5]. However, a weakness of the MDGs monitoring was an insufficient attention to water safety [1,6], which became a key element of the target task for water supply and sanitation upon design of the Sustainable Development Goals (SDG 6).

According to the United Nations Resolution 64/292: “The human right to water entitles everyone to sufficient, safe, acceptable, physically accessible and affordable water for personal and domestic uses” [2,7]. Therefore, SDG 6.1 call for full coverage of safely managed drinking water by 2030. The “Safely managed drinking water” indicator includes the three following conditions: accessible on premises, available when needed and free from contamination [8,9].

This goal is a huge challenge for all countries, not only for low- and middle-income ones [10]. The commitment to “leave no one behind” requires a focus on rural areas, which is typically neglected [4,11,12,13]. About 844 million people on Earth do still not have access to basic water supplies and 79% of them are rural residents [14]. At the same time, 2.1 billion people have no safely managed drinking water supply system service. This means that 14.9% of the urban- and 45.2% of the rural population need improved services [9].

A person needs 50 to 100 litres of water per day to meet physiological and hygienic needs [15,16,17]. People facing a limit of 20 litres per capita per day will therefore be exposed to a high level of health concerns. Rural residents usually live in worse economic conditions than urban ones and this affects the volume of water use [18,19].

Kazakhstan is one of the countries on the Eurasian continent that experiences the most severe water shortages. Water shortage and its poor quality have been determined as vital issues threatening the future prosperity of the country [20,21,22]. Furthermore, in Kazakhstan the coverage of water supply in the urban and rural areas differ significantly. Approximately 90% of urban people have access to safely managed drinking water, whereas in the rural areas this rate is only 28% [5,23]. Therefore, rural areas constitute the greatest challenge in the efforts to provide safe water for all.

The objective of the paper is to assess the current access to and the perceived water quality in the villages with various types of water supply. Although official statistics on water access per person in each village are available, that do not reflect the complex realities of the current situation. Therefore, a questionnaire survey was carried out in villages in the central part of Kazakhstan to illustrate this complexity and the obtained data was compared with the official one. The factors affecting the volume of water consumption and preferences to use alternative sources among centralized water supply users were identified. In addition, people’s satisfaction with the quality of drinking water and the reliability of different services were evaluated.

## 2. Materials and Methods

### 2.1. Source Description

**Figure 1:**
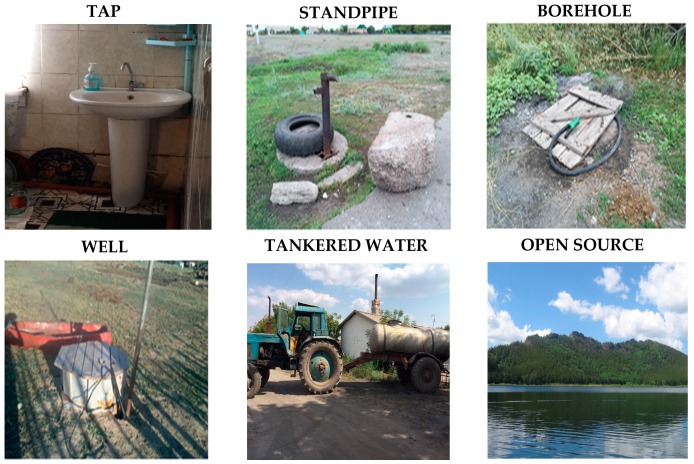
Sources of drinking water.

Drinking water is domestic water used for both drinking and hygiene purposes [24]. It can be supplied from different sources. Figure 1 shows the six available sources for such water in Kazakhstan. Centralized water provision is distributed through taps and standpipes, with water supplied from either surface or groundwater and this water is usually treated. Standpipes are provided along the pipelines at specified intervals. However, tap water inside a house is available only at the expense of a house owner. The government provides the centralized water supply, therefore the local administrative authority shall regularly check it for the presence of contaminants. Decentralized water supplies from boreholes and wells do not have any delivery services to houses and can be used public or individual. A permit for drilling new boreholes and wells is provided by the local administrative authority based on prior investigation of the field. They are also intended to do regular water quality tests throughout the operation period. However, the population sometimes use unregistered boreholes and wells, which means no control by the local administrative authority. Other sources of drinking water, such as tankered water and water from open sources, are not considered safe. However, due to the absence of water supply alternatives tankered water is included in official statistics and is regarded as a makeshift measure for drinking water supply provision for the population. Water is delivered to villages in a tanker, usually once a week and people pay for each litre on site. A company selected by the local administrative authority is responsible for a timely delivery and for the quality of water. Finally, an open source can be a spring, river or lake. They are completely absent in the official statistics and are utilized by individuals [25].

Rural people have to use multi-sources due to the lack of a stable water supply system in the villages. Households usually classify them based on their purpose for using water [26]. For instance, tap water for drinking, wells for hygiene, rainwater and thawed water for garden irrigation, etc.

### 2.2. Area Description

The study was carried out in the Bukhar-Zhyrau district (49°57.21′ N–73°43.01′ E, 500–700 m elevation, 14,576 km^2^), located in the central part of Kazakhstan. The climate is continental with an average temperature of +19 to +21 °C in July and −15 to −17 °C in January, in addition to an average annual precipitation of 300–350 mm. The topography is flat and most of the territory of the district is covered by the Kazakh Uplands. A population of 64,683 (in 2017) live in 67 villages scattered throughout the region [27].

Groundwater is the main water resource and the population is provided with various types of water supply. Centralized piped water supply is used by 51,752 people, including 6083 standpipe users and 45,669 in-house water conduit users. Decentralized water supply is used by 12,431 people, including 9001 borehole users and 3430 well users. Finally, tankered water is used by 500 people [27]. To make a complete pattern of basic advantages and disadvantages of water supply in the region under study four villages, each with the largest percentage of users of one of the three types of water supply, were selected for further investigation (Table 1): Botakara with mixed (both centralized and decentralized), Dubovka and Karazhar—centralized, and Asyl—tankered water supply.

### 2.3. Questionnaire Development

The questionnaire was developed based on the findings of a pilot study conducted by Tussupova et al. [28,29] in the Kazakh and Russian language, since both Kazakh and Russian speakers reside in the region under study. An ethical approval was obtained from the Bioethics Committee (Karaganda State Medical University, Karaganda, Kazakhstan, Protocol #110 of 17.10.2016) and the questionnaire was accepted during a session of the Scientific Evaluation Committee (Karaganda State Medical University, Karaganda, Kazakhstan, Protocol #6 of 14.06.2017). This study was approved and verified by the local administrative authority of Bukhar-Zhyrau district. The respondents were aware that participation therein was voluntary and that they could renounce providing any information at any time without reasons. All the persons polled signed an informed data collection consent statement.

The aim of the questionnaire was to assess what sources were used by the rural population and their satisfaction with the quality and quantity of the drinking water supply. The questionnaire covered the following topics: type of source mostly used for drinking purposes, reasons for searching for other water sources despite having a tap at home, volume of water consumption, time spent on water collection, additional purchase of bottled water, household water treatment methods, perceived quality and reliability of water supply systems.

### 2.4. Sample Collection

#### 2.4.1. Calculation of Sample Size

The survey was carried out during July-December 2017. First, the official data provided by the local administrative authority for information about water supply systems available in the given region was studied. Then 1369 randomly selected households in four villages were interviewed. Finally, the obtained data was analysed aided with STATISTICA 13.3 (StatSoft, Tulsa, OK, USA) software. The sample size was calculated using the following formula [30]:

$$n=\frac{p\times q\times Z_{\alpha }^{2}\times N}{{∆}^{2}\times N+p\times q\times Z_{\alpha }^{2}}$$

where *n* is the required sample size; *p* and *q* is a part and its inverse value in each class of the general totality (*p* = 0.5; *q* = 0.5); *Z_α_* is a constant (set by convention according to the accepted *α* error and whether it is a one-sided or two-sided effect) as shown on Table 2:

*N* is general totality amount (*N_1_* = 6252; *N_2_* = 4114; *N_3_* = 1035; *N_4_* = 294); ∆—the difference in effect of two interventions which is required (estimated effect size) (∆ *= 5%*):

$$\begin{array}{cc} n_{1}=\frac{50\times 50\times 1.96^{2}\times 6252}{5^{2}\times 6252+50\times 50\times 1.96^{2}}=362; & n_{2}=\frac{50\times 50\times 1.96^{2}\times 4114}{5^{2}\times 4114+50\times 50\times 1.96^{2}}=353;\\ n_{3}=\frac{50\times 50\times 1.96^{2}\times 1035}{5^{2}\times 1035+50\times 50\times 1.96^{2}}=280; & n_{4}=\frac{50\times 50\times 1.96^{2}\times 294}{5^{2}\times 294+50\times 50\times 1.96^{2}}=167. \end{array}$$

Provided inevitable loss amongst the participants in the course of the study (for various reasons), the calculated sample size was increased by 20%:

$$\begin{array}{cc} n_{1}=362+\left(20\%\times n_{1}\right)=434; & n_{2}=353+\left(20\%\times n_{2}\right)=424;\\ n_{3}=280+\left(20\%\times n_{3}\right)=336; & n_{4}=167+\left(20\%\times n_{4}\right)=200. \end{array}$$

In the course of questionnaire survey 25 persons resigned from the investigation: four from Botakara; three from Dubovka; seven from Karazhar, and 11 from Asyl. Thus, the total number of the respondents was 1369 instead of 1394.

#### 2.4.2. Calculation of Water Consumption

Those households that use the tap pay for each m3 of water according to the meter readings. The respondents indicated the volume of water consumption (*x*) according to the payment receipts for the last month. When analyzing, water consumption per person per day (L) was calculated by the following formula:

$$Water\;consumption\;per\;person\;per\;day\;\left(L\right)=\frac{x\;\left(m^{3}\right)\times 1000\left(l\right)}{number\;of\;people\;in\;the\;house\times 30\;\left(days\right)}$$

Households that use sources without any delivery services collect and store water in tanks. During the interview, the respondents indicated the volume of tanks (*x*) and how often they had to fetch water. According to the findings, water consumption per person per day (L) was calculated as follows:

$$Water\;consumption\;per\;person\;per\;day\;\left(L\right)=\frac{x\;\left(l\right)}{number\;of\;people\;in\;the\;house\times number\;of\;days\;of\;use}$$

### 2.5. Description of Respondents

The questionnaire included the answers of one family member over 18 years who was responsible for water use from each household. The overall burden of collecting and using water in population is usually much higher in women than in men [31]. Our results have also confirmed this fact, since 63% of respondents were women and the remaining 37% were men. The respondents were between 19–70 years old. On average, 80% of them had lived in the studied villages from birth and each household included one to nine persons. Since the selection of the households was randomized, the level of education within the communities surveyed was not specifically studied.

## 3. Results

### 3.1. Villages with Access to Tap Water

Comparing the official data from Table 1 and the collected data from Table 3, it was found that the residents often used alternative water sources, even though they were provided with tap water supply. According to official data, 42.55% of the population of Botakara village had a water pipe in a house and 7% of them used standpipes outdoors, but only 25.35% of the respondents indicated taps as a source of drinking water and 51.44%—standpipes. In addition, 34.49% of the villagers had registered boreholes, and 15.96% had registered wells in their yards. Nevertheless, our data showed that only 16.51% and 6.74% used this kind of sources.

The situation was different in Dubovka village. There, 100% of the population was provided with centralized water supply and 98.06% of them had water taps inside their houses (Table 1). However, nearly half of the respondents indicated alternative water points as a source of drinking water due to the time limited water service (Table 3). Private unregistered boreholes and wells were used by 23.52% and 17.34% of the respondents, respectively. Moreover, 15.44% of villagers preferred to use water from natural open sources.

A similar situation was observed in Karazhar village. According to the data in Table 1, 100% of the population was provided with centralized water supply. Nevertheless, as many as about 78% of the respondents indicated other water sources: 28.57% had unregistered boreholes, 31.31% unregistered wells and 18.24% independently brought water from natural open sources (Table 3). The central water supply in the village was served all year round on a scheduled basis, four hours in the morning and three in evening. According to the respondents’ description, tap water was muddy. Therefore, people had to let water run for a long time, as well as to settle and boil it before each use.

**Figure 2:**
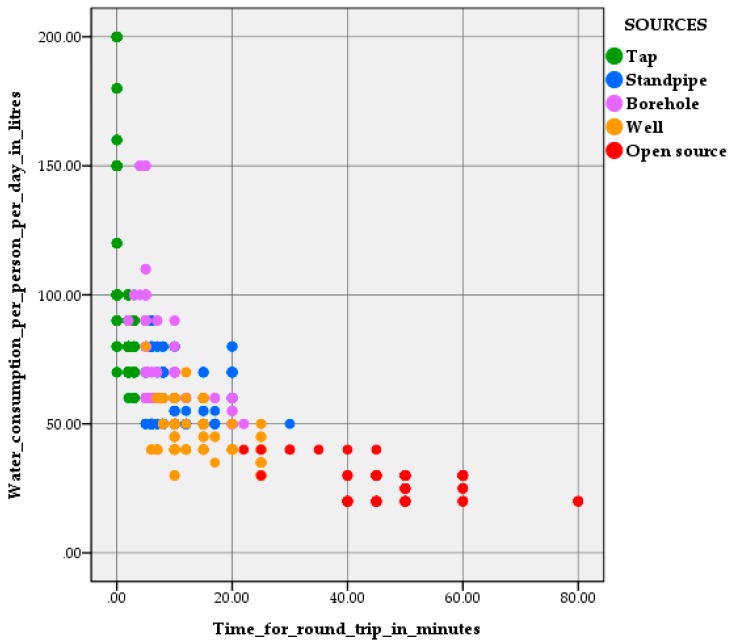
Water consumption in terms of a water supply source used by households and the time spent on water collection.

The amount of used water depended on a source of water supply used by households and the time required to transport water from a source to a house. The linear regression between the volume of water consumption, a water supply source and the time spent on water collection was moderately downhill (R = −0.633; *p* = 0.01) (Figure 2). This relationship showed that in 99% of cases with increasing time of water transporting, its consumption decreased. A type of water source and the time of water transportation to a house explained 40% of the variation in water consumption among the respondents, the remaining 60% of the variation was caused by influence of other unaccounted factors.

**Figure 3:**
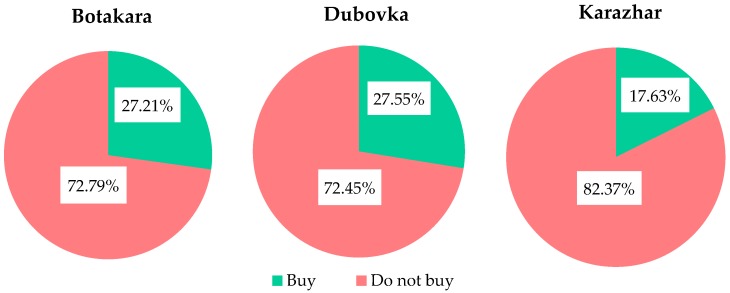
Additional purchase of bottled water.

As shown in Figure 3, 27.21% of the respondents in Botakara, 27.55% in Dubovka and 17.63% in Karazhar bought bottled water. However, Karazhar village differed from the other two in the frequency and quantity of buying bottled water. In Botakara and Dubovka 50% of people who bought bottled water did this irregularly, while in Karazhar villagers had to purchase it two or three times a week. In the first two villages, residents bought average 4.18 and 4.71 litres at a time respectively. In Karazhar this number was 6.2 litres.

**Figure 4:**
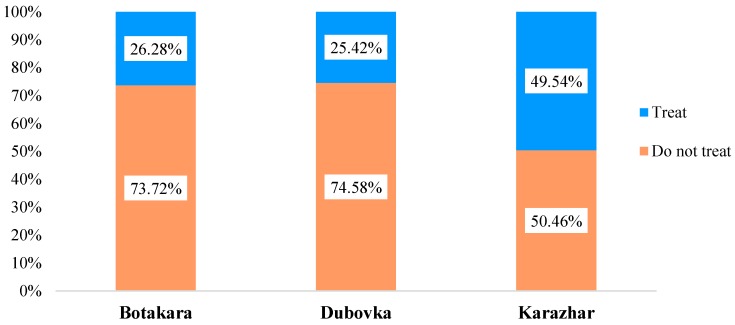
Use of household water treatment methods in the villages.

Some households treated drinking water at household level (Figure 4). In Karazhar 49.54% of the respondents used some methods of household treatment, while this number in Botakara and Dubovka was 26.28% and 25.42% respectively. For this treatment, 76.07% of the respondents who purified water in Karazhar said that they used a factory filter. More than half of them changed a filter once a month and spent an average of 885 tenge (US $2.48 as on August 31, 2018) on each piece.

Multiple p-level comparisons by the Kruskal-Wallis test showed that water from taps in houses, outdoor standpipes and boreholes was no different in satisfaction with the quality of drinking water and reliability of sources according to the respondents (Table 4). Quality and reliability are not independent factors. System breakdown impacts both quantity and quality, as the water is frequently of poor quality after such an event. Thus, *reliability* was essentially a measure of how often there was a problem concerning the delivery of water of an acceptable quality. In Dubovka and Karazhar villages, there were statistically significant differences in the quality indicators of water taken from wells and open sources, in contrast to water from the sources mentioned above. The villagers in Dubovka who used wells and open sources were not satisfied with its quality and reliability, as they rated them as “poor” (81% and 71.73% respectively) and “unreliable” (94.06% and 86.7% respectively). Almost the same situation was observed in Karazhar: 66.87% of villagers were not satisfied with the quality of water from wells and 74.77% from open sources. Also, 76.6% and 85.11% of the respondents considered the use of wells and open sources respectively to be unreliable.

**Figure 5:**
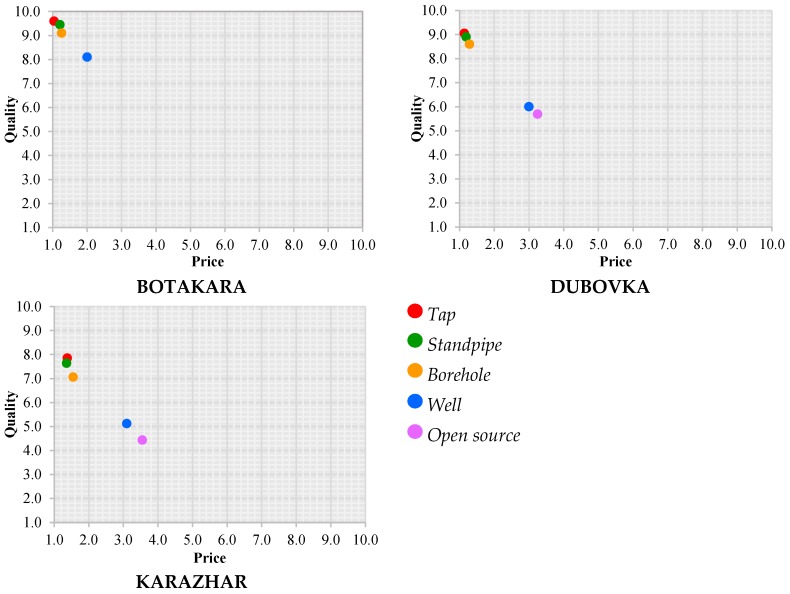
Subjective assessment of quality-price ratio on drinking water by the respondents.

Figure 5 shows the subjective assessment of the price and quality of drinking water given by the respondents depending on a used water source on a scale from one to ten. They stated the quality of drinking water in points in accordance with their impression, where one point was low and ten points was good quality. The price was converted into points based on the impression of the cost of drinking water, where one point was acceptable and ten points was expensive. The ratio of quality and price was calculated as follows:

$$Quality-price\;ratio=\frac{Quality}{Price}$$

The residents of Botakara gave a high estimate in comparison with the other two villages; the estimates in Karazhar were very low (not above 5.7 points for taps and standpipes). The assessment given by the villagers fell depending on a used water source in the following sequence: tap > standpipe > borehole > well > open source. In most cases, people believed that the costs of an agreement with a third party for drilling a well as well as independent water transportation from open sources did not conform to water quality. This number for water from wells in Botakara was estimated at 4.14 points, in Dubovka at 2 points and in Karazhar at 1.7 points. The residents of the last two villages also used open sources and rated them at 1.97 and 1.35 points respectively.

### 3.2. Villages with Tankered Water

In Kazakhstan, a number of villages have an acute water shortage due to the lack of sources in their territory. It is estimated that the economic condition of the villages is poor. The population is provided with limited volumes of tankered water, the quality of which is doubtful. At the time of the study, in the Bukhar-Zhyrau district, there were four similar villages. One of them was Asyl, where 294 people lived. All people there used tankered water. The distance of water delivery was 17 km from a water source. 

In Asyl village, the collected data coincided with the official ones, but the reason was the absence of alternative source of drinking water supply in the territory. There was only one tanker for the whole settlement, which brought water once a week according to the schedule (every Friday at midday local time). Therefore, when the transport broke down, the population had no drinking water for two–four weeks. Water tankers must be cleaned and disinfected before use at least once every three months [32]. According to the interview with the driver, this requirement was not always met.

The average water consumption in the village was 41.67 litres per person per day. Some residents stated that they spent an average of 103 minutes (for round trip) for self-delivery of water from alternative sources to a house. The data showed that 68.78% of the respondents bought bottled water as needed for drinking and cooking only. In case of water shortage or lack of delivery, most villagers used rainwater and thawed water for hygiene purposes.

In the village 44.44% of residents indicated that they regularly treated drinking water at home, 24.87% of them boiled water before consumption, and 67.72% used a factory filter. However, the issue was that the population did not know how to operate it properly. This was evident from the fact that 50.26% of those who used filters at home had not changed them it from the moment of purchase.

In Asyl village, the level of satisfaction with the quality of water and reliability of the source was very low (Table 5). Since 77.78% of residents believed that, its quality was “poor”, and 98.94% estimated the reliability of tankered water supply as “unreliable”. Furthermore, villagers considered that the price of tankered water was not in line with its quality. They rated it at only 2 points. 

## 4. Discussion

Tap water installed in villages by the government was not able to fully satisfy the populations’ drinking water demands. There had been some constant interruptions in the systems due to technical problems, which in turn worsened the quality of the supplied water. The quality was further reduced, because the population had underused the system’s capabilities [33,34,35]. Even though villagers were provided with tap water by the government, significant numbers used water from alternative sources of an unknown quality. When analyzing the reasons that led to this situation, it turned out that respondents most often indicated in the questionnaire the following: doubts regarding the quality of tap water; use of other sources by habit, as they were accustomed to it during water scarcity; and availability of cheaper or free water sources. The villagers also explained that scheduled water supply was the reason for searching for other water sources despite having a tap at home. This was especially the case during summer time, when water consumption increased due to garden irrigation.

Another problem concerned the quality of water supply for the residents from unregistered boreholes and wells in the villages. These boreholes and wells were not tested for compliance with the sanitary standards before and during the operation. Due to acute water supply shortage, the population also had to use water from open sources; brackish water from underground sources recommended only for domestic purposes as well as rain and thawed water. This situation was regarded as highly unsatisfactory.

A study of the water use characteristics was greatly significant for a sustainable development of rural regions, especially in countries with a deficiency of water resources. The more time people spent on water transportation from a source to a house, the less water they consumed to the detriment of their physiological and hygienic needs. Moreover, the amount of water used dropped sharply with decreased quality or inconvenience related to a source of water supply used by households. 

Water consumption among taps, standpipes and boreholes users was found to be 50 to 200 litres per person per day, while this number among open sources and tankered water users did not reach 50 litres per day. Other factors affecting the amount of water consumption included religious obligations, water price, family income and climate condition, as well as relations and intentions in regard to preservation of water resources [36,37,38,39].

The population considered additional purchase of bottled water and treating water at home to be desperate measures. Bottled water was needed in periods of acute water shortage, when percentage of purchase was especially high in the village with tankered water. Water was treated at home in villages where residents doubted the water quality and took responsibility for its additional treatment. People who were the most satisfied with the quality of used drinking water and reliability of sources lived in Botakara, because they did not use water from open sources, and it was in this area where the majority of boreholes and wells had been registered. The less satisfied people lived in Dubovka and Karazhar due to low quality of water from wells and open sources, and in Asyl because of tankered water. People gave a poor estimate to reliability of these sources, although they still consumed the water from them. 

In spite of the fact that the government tries to provide rural regions with tap water supply, the study has revealed various challenges in this endeavour. It is necessary to find a balance between the quantity and quality of water. In villages where there is a need to prioritize access to sufficient water quantity, the water consumption can be increased by means of timely repair and maintenance of the system, which is in turn a guarantee of uninterrupted supply of drinking water. In villages where the water quality is the dominant factor, priorities should be directed to appropriate drinking water treatment methods and training to encourage the population to choose the right water source. To this end, there should be an emphasis on making the healthy benefits of tap water associated with its high microbiological quality widely known. Moreover, to reduce the stress on limited water resources, there is a need for a more effective management and implementation of water preservation measures as well as improvement of the technical conditions of water supply lines, and sewage facilities. There is also a need for efficient and hygienic water use training for the population.

The villagers thought that the costs of an agreement with a third party for drilling a well and independent water transportation from open sources as well as the price of tankered water were not in line with its quality. Even while there was one source of water for taps and standpipes in each village, satisfaction with its quality and reliability varied due to technical problems in water supply plants. Upon their assessment of the price and quality of drinking water subject to the water source used, the respondents gave more points to tap water than to standpipe in all villages under investigation. This was because in this case they estimated the quality of the water as well as the convenience service. Obviously, water from the centralized system cannot be considered to be safe as long as users occasionally prefer other, uncontolled sources.

## 5. Conclusions

Decentralization of water management, monitoring of both water supply and water use and a tailor-made approach to each village are necessary to achieve the Sustainable Development Goals objective of providing rural people with safely managed drinking water. Providing safe water supply to rural Kazakhstan will be a tremendous challenge that the government needs to tackle as soon as possible.

It is only in cooperation with the local community, government bodies can identify systemic sustainability problems, and develop and implement policies for water access in premises; water that is available as needed and free from contamination. This cooperation will also ensure sustainable public health and bring economic benefits to villages. Consequently, this analysis of consumer demand on the existing water supply systems in the villages and people’s preferences in choosing the source of drinking water can contribute to more effective water supply planning and, thereby, support a sustainable development of rural regions. 

## Figures and Tables

**Table 1:** Number of population in investigated villages by type of water supply according to the official data and the sample size.

Types of Water Supply	Villages	Botakara (1)	Dubovka (2)	Karazhar (3)	Asyl (4)
CENTRALIZED	tap	2660	4034	650	−
	standpipe	438	80	385	−
	∑	3098	4114	1035	−
DECENTRALIZED	borehole	2156	−	−	−
	well	998	−	−	−
	∑	3154	−	−	−
TANKERED	∑	−	−	−	294
SAMPLE SIZE		362	353	280	167
SAMPLE SIZE + 20%		434	424	336	200
SURVEYED HOUSEHOLDS		430	421	329	189

**Table 2:** Critical values of Z for standardized normal distribution.

α Error	0.005	0.01	0.012	0.02	0.025	0.05	0.1	0.15	0.2	0.25	0.3
one-sided	2.567	2.326	2.257	2.054	1.96	1.645	1.282	1.036	0.842	0.674	0.524
two-sided	2.807	2.576	2.513	2.326	2.242	1.960	1.645	1.440	1.282	1.150	1.036

**Table 3:** Percentage of respondents by the drinking water sources according to the collected data.

Types of Water Supply	Villages	Botakara (1)	Dubovka (2)	Karazhar (3)
CENTRALIZED	tap	25.35%	28.5%	15.5%
	standpipe	51.44%	15.2%	6.38%
	∑	76.79%	43.7%	21.88%
DECENTRALIZED	borehole	16.51%	23.52%	28.57%
	well	6.7%	17.34%	31.31%
	∑	23.21%	40.86%	59.88%
Open source	∑	0%	15.44%	18.24%

**Table 4:** Level of satisfaction with the quality of used drinking water and reliability of sources according to the respondents’ assessment.

Villages		Botakara (1)				Dubovka (2)					Karazhar (3)				
Sources of Water Supply		Tap	Standpipe	Borehole	Well	Tap	Standpipe	Borehole	Well	Open Source	Tap	Standpipe	Borehole	Well	Open Source
SATISFACTION LEVEL = Turbidity + Odor + Taste	good	65.35%	70.7%	86.74%	46.28%	81.24%	60.1%	51.07%	0%	1.66%	33.74%	46.47%	19.45%	2.13%	0%
	average	27.91%	28.84%	10.23%	53.72%	4.51%	18.05%	31.83%	19%	26.6%	36.78%	53.19%	60.49%	31%	25.23%
	poor	6.74%	0.47%	3.02%	0%	14.25%	21.85%	17.1%	81% ^1^	71.73% ^1^	29.48%	3.34%	20.06%	66.87% ^1^	74.77% ^1^
RELIABILITY	reliable	42.33%	43.49%	76.51%	42.79%	78.62%	39.9%	52.26%	0%	0%	17.63%	28.57%	13.07%	0%	0%
	not always	50%	54.65%	17.67%	57.21%	0%	26.6%	21.62%	5.94%	13.3%	35.26%	14.29%	64.13%	23.4%	14.89%
	unreliable	7.67%	1.86%	5.81%	0%	21.38%	33.49%	26.13%	94.06% ^1^	86.7% ^1^	47.11%	57.14%	22.8%	76.6% ^1^	85.11% ^1^

^1^ Significant at *p* < 0.05.

**Table 5:** Level of satisfaction with the quality and reliability of tankered water supply according to the respondents’ assessment.

Village		Asyl (4)
Source of Water Supply		Tankered Water
SATISFACTION LEVEL = Turbidity + Odor + Taste	good	6.88%
	average	15.34%
	poor	77.78%
RELIABILITY	reliable	0%
	not always	1.06%
	unreliable	98.94%
